# Osr1 Interacts Synergistically with Wt1 to Regulate Kidney Organogenesis

**DOI:** 10.1371/journal.pone.0159597

**Published:** 2016-07-21

**Authors:** Jingyue Xu, Han Liu, Ok Hee Chai, Yu Lan, Rulang Jiang

**Affiliations:** 1 Division of Developmental Biology, Cincinnati Children’s Hospital Medical Center, Cincinnati, OH 45229, United States of America; 2 Department of Anatomy, Chonbuk National University Medical School and Institute for Medical Sciences, Deokjin-gu, Jeonju 561–756, Republic of Korea; 3 Division of Plastic Surgery, Cincinnati Children’s Hospital Medical Center, Cincinnati, OH 45229, United States of America; UCL Institute of Child Health, UNITED KINGDOM

## Abstract

Renal hypoplasia is a common cause of pediatric renal failure and several adult-onset diseases. Recent studies have associated a variant of the *OSR1* gene with reduction of newborn kidney size and function in heterozygotes and neonatal lethality with kidney defects in homozygotes. How OSR1 regulates kidney development and nephron endowment is not well understood, however. In this study, by using the recently developed CRISPR genome editing technology, we genetically labeled the endogenous Osr1 protein and show that Osr1 interacts with Wt1 in the developing kidney. Whereas mice heterozygous for either an *Osr1* or *Wt1* null allele have normal kidneys at birth, most mice heterozygous for both *Osr1* and *Wt1* exhibit defects in metanephric kidney development, including unilateral or bilateral kidney agenesis or hypoplasia. The developmental defects in the *Osr1*^*+/-*^*Wt1*^*+/-*^ mouse embryos were detected as early as E10.5, during specification of the metanephric mesenchyme, with the *Osr1*^*+/-*^*Wt1*^*+/-*^ mouse embryos exhibiting significantly reduced Pax2-positive and Six2-positive nephron progenitor cells. Moreover, expression of *Gdnf*, the major nephrogenic signal for inducing ureteric bud outgrowth, was significantly reduced in the metanephric mesenchyme in *Osr1*^*+/-*^*Wt1*^*+/-*^ embryos in comparison with the *Osr1*^*+/-*^ or *Wt1*^*+/-*^ littermates. By E11.5, as the ureteric buds invade the metanephric mesenchyme and initiate branching morphogenesis, kidney morphogenesis was significantly impaired in the *Osr1*^*+/-*^*Wt1*^*+/-*^ embryos in comparison with the *Osr1*^*+/-*^ or *Wt1*^*+/-*^ embryos. These results indicate that Osr1 and Wt1 act synergistically to regulate nephron endowment by controlling metanephric mesenchyme specification during early nephrogenesis.

## Introduction

Renal hypoplasia, defined as abnormally small kidney with normal morphology and reduced nephron number, is a common cause of congenital kidney failure and a significant risk factor for hypertension or chronic renal failure in adults [1–3]. The molecular mechanisms that determine nephron number are still not well understood, however. In mammals, three distinct types of kidney structures develop bilaterally during embryogenesis along the anterior-posterior body axis: the pronephroi, which form in the anterior intermediate mesoderm (IM) and regress quickly but the nephric duct continues to extend posteriorly to induce subsequent kidney development; the mesonephroi, which are structurally more complex but are also transient during midgestation; and the metanephroi, which continue morphogenesis from midgestation through perinatal stages and function as the blood filters throughout postnatal life. In mice, metanephric kidney development initiates around embryonic day 10 (E10) with the establishment of a unique population of nephrogenic cells, called metanephric mesenchyme (MM), in the posterior IM. The MM induces outgrowth of the ureteric bud (UB) from the nephric duct at the level of hindlimb buds. The UB invades into MM and induces MM cells to condense around the UB tip, forming the cap mesenchyme (CM). As development proceeds, the CM induces UB to branch repeatedly and a subset of CM cells in the armpit of each new branch undergo mesenchymal-epithelial transformation to form a renal vesicle, which subsequently differentiates into a nephron. All nephrogenic progenitor cells in the metanephric kidney are depleted by the final wave of nephrogenesis in the perinatal period and no new nephron formation initiates thereafter [4].

Prior to UB outgrowth, the MM expresses a unique combination of signaling molecules and transcription factors, including the glial derived neurotrophic factor (Gdnf) and the transcription factors Eya1, Pax2, Six1, and Six2 [5]. Gdnf is the major signal for UB induction, acting through its receptors Ret and Gfra1 in the nephric duct epithelium. Mice lacking *Gdnf*, *Ret*, or *Gfra1*, fail to form the UB and die perinatally with bilateral renal agenesis [6–11]. Genetic down-regulation of the Gdnf/Ret signaling pathway results in significantly reduced ureteric bud branching, nephron number and kidney size, which are observed in the *Gdnf* ^+/-^ mice and *Ret*^*Y1062F*^ knockin homozygous mice [12–14]. The Eya1, Pax2, and Six1 transcription factors are each required for activation and/or maintenance of *Gdnf* expression in the metanephric mesenchyme and mice lacking any one of them die perinatally with bilateral renal agenesis [15–17]. Mutations in *EYA1*, *PAX2*, *SIX1*, or *SIX2*, have been found in a subset of human patients with renal agenesis or hypoplasia [18–22]. A frameshift mutation in *Pax2* also resulted in renal hypoplasia in heterozygous mice, which correlated with elevated apoptosis in the UB epithelium [23]. Mice lacking Six2 function exhibited severe renal hypoplasia due to premature differentiation and rapid depletion of nephron progenitor cells following initial UB branching [24]. These results indicate that MM or UB cell survival, the reciprocal interactions between the MM and UB epithelium, and the balance between progenitor maintenance and differentiation, all play important roles in controlling the nephron number.

The *odd-skipped related 1* (*Osr1*) gene encodes a homolog of the *Drosophila* odd-skipped zinc finger protein [25, 26]. *Osr1* expression is first activated in the nascent IM at the late gastrula stage (E7.5) during mouse embryogenesis [27]. Strong *Osr1* expression persists in the nephrogenic mesenchyme but is completely down-regulated upon mesenchymal-epithelial transition into the nephric duct or renal vesicles during kidney development [27, 28]. Genetic lineage tracing studies demonstrated that *Osr1*-expressing IM cells give rise to the majority of cell types in the kidney but *Osr1* expression itself undergoes progressive restriction to the CM cells during metanephric kidney organogenesis [29]. In *Osr1*^*-/-*^ mutant mouse embryos, the nephric duct formed and extended to the posterior IM, but no morphologically distinguishable MM was detected and the nephrogenic mesenchyme cells exhibited aberrant apoptosis from E9.5 to E10.5, indicating that Osr1 function is required for early nephrogenic mesenchyme cell survival [27, 28]. Through tissue-specific genetic analysis, we showed that Osr1 also plays a critical role in maintaining the balance between progenitor cell renewal and nephron differentiation during metanephric kidney organogenesis [30]. Recently, a single nucleotide variant in an exonic splice enhancer in the *OSR1* gene was associated with reduction in newborn kidney size and function in humans [31]. In addition, homozygosity of the same variant *OSR1* allele was associated with neonatal lethality with congenital kidney defects [32]. In this report, we demonstrate that Osr1 interacts synergistically with another important kidney developmental regulator Wt1 [33] to control nephron endowment through regulation of MM specification.

## Results

### Osr1 and Wt1 are co-expressed in the metanephric mesenchyme and form protein interaction complexes

**Fig 1:**
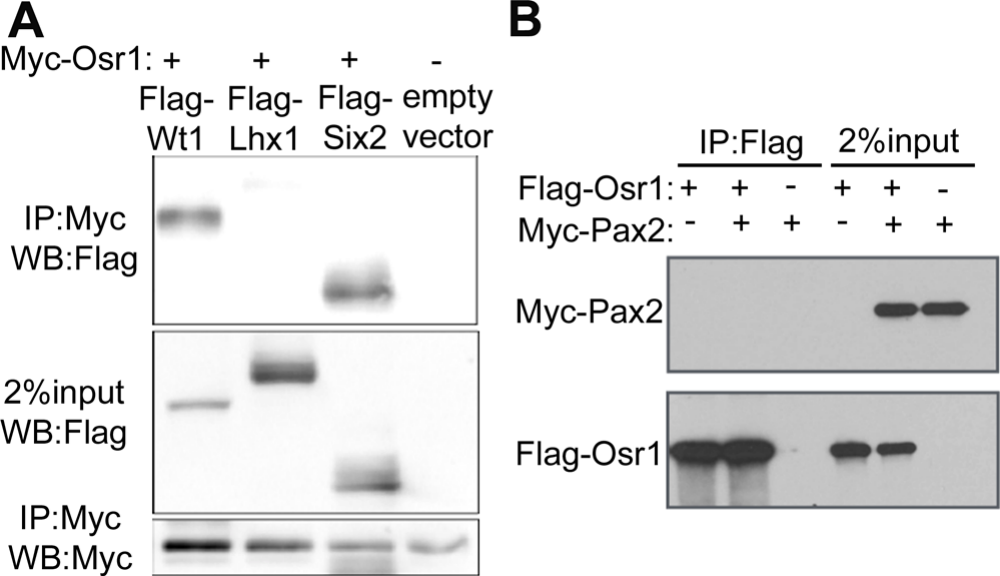
Osr1 and Wt1 form protein interaction complex when co-expressed. (A) HEK293T cells were co-transfected with plasmids expressing Myc-tagged Osr1 and Flag-tagged Wt1, Lhx1, Six2, or a Flag-tag vector, respectively. Immunoprecipitation was carried out with an anti-Myc antibody, and the resulting protein complexes resolved by western blotting and detected with an anti-Flag antibody. Note that Flag-Wt1 and Flag-Six2 were co-immunoprecipitated with Myc-Osr1. (B) HEK293T cells were co-transfected with plasmids expressing Flag-tagged Osr1 and Myc-tagged Pax2. Immunoprecipitation was carried out with an anti-Flag antibody, and the resulting protein complexes resolved by western blotting and detected with an anti-Myc antibody.

We first co-expressed Myc or Flag-tagged Osr1 with each of several transcription factors, including Wt1, Lhx1, Six2 and Pax2, which are all co-expressed with Osr1 at some point in the developing metanephric mesenchyme [24, 30, 33–37], in HEK293T cells and performed co-immunoprecipitation assays (Fig 1). We showed previously that Osr1 and Six2 interact with each other in maintaining nephron progenitor cells during metanephric kidney development, thus the Osr1-Six2 interaction provides a positive control [30]. Immunoprecipitation of Myc-Osr1 pulled down Flag-Wt1, but not Flag-Lhx1, from the co-transfected cells (Fig 1A). Immunoprecipitation of Flag-Osr1 did not pull down myc-Pax2 from the co-transfected cells (Fig 1B). These results indicate that Osr1 could form interactive protein complexes with Wt1 when co-expressed.

**Fig 2:**
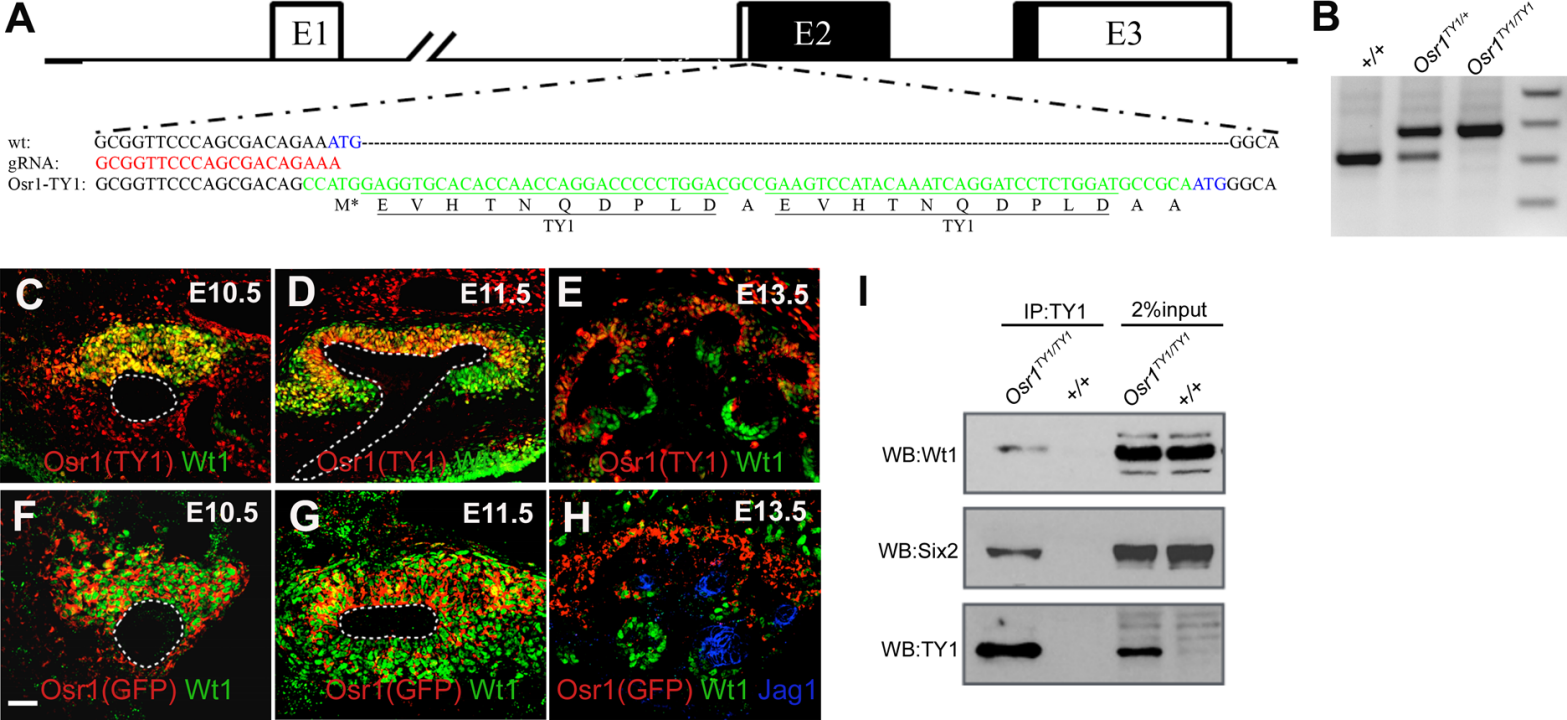
Osr1 and Wt1 are co-expressed in the early metanephric mesenchyme during kidney development and physically interact *in vivo*. (A) Schematic diagram of the *Osr1* gene structure and strategy for CRISPR/Cas9-mediated insertion of the 2xTY1 epitope tag at the N-terminus of the endogenous Osr1 protein. The gRNA target sequence is shown in red font. The sequence of the mid-portion of the oligonucleotide donor template for homology directed repair contains the 2xTY1 tag-coding sequence (shown in green font). The endogenous *Osr1* ATG codon is shown in blue font. The tag sequence contains its own ATG initiation codon at the 5’ end (indicated by M* underneath the sequence). (B) *Osr1*^*TY/+*^and *Osr1*^*TY1/TY1*^ mice were identified by PCR genotyping. (C-E) Immunofluorescent staining for the TY1 epitope (red) and the Wt1 protein (green) on sections of E10.5 (C), E11.5 (D), and E13.5 (E) *Osr1*^*TY1/+*^ embryos. (F-H) Immunofluorescent staining for eGFP (red), Wt1 (green) and Jag1 (blue) on sections of E10.5 (F), E11.5 (G), and E13.5 (H) *Osr1*^*GCE/+*^ embryos. The ureteric bud is outlined with white dashed circle in (C-H). (I) E16.5 kidneys were collected from *Osr1*^*TY1/TY1*^and wildtype embryos. Immunoprecipitation was carried out with an anti-TY1 antibody, and the resulting protein complexes resolved by western blotting and detected with anti-Six2 and anti-Wt1 antibodies. Scale bar, 50 μm.

To validate that Osr1 interacts with Wt1 during kidney development *in vivo*, we used the recently developed CRISPR (clustered regularly interspaced short palindromic repeat)/Cas9 (CRISPR-associated) genome editing system [38, 39] to insert a 2xTY1 epitope tag at the N-terminus of the endogenous Osr1 protein (Fig 2A). The *Osr1*^*TY1/+*^ CRISPR founder mice were crossed to wildtype C57BL/6J mice and the G1 *Osr1*^*TY1/+*^ hemizygous mice were identified by PCR genotyping and verified by Sanger sequencing. Intercrossing of the *Osr1*^*TY1/+*^ hemizygous mice produced *Osr1*^*TY1/TY1*^ homozygous mice, which were born at Mendelian ratio and did not display any phenotypic difference from wildtype littermates. We further crossed *Osr1*^*TY1/TY1*^ homozygous mice to *Osr1*^*+/-*^ mice heterozygous for a targeted null Osr1 allele [27] and found that the *Osr1*^*TY1/-*^ mice survive and breed normally, indicating that the inframe fused TY1 tag did not affect the Osr1 protein function.

We compared the expression pattern of TY1-Osr1 from *Osr1*^*TY1*^ allele and that of the endogenous Wt1 protein in kidney development in *Osr1*^*TY1/TY1*^ embryos at E10.5, E11.5, and E13.5, respectively (Fig 2C–2E). We found that TY1-Osr1 fusion protein was co-expressed with Wt1 in the metanephric mesenchyme cells at E10.5 and E11.5 (Fig 2C and 2D). At E13.5, TY1-Osr1 was expressed at high levels in the undifferentiated cap mesenchyme but is absent from the renal vesicles (Fig 2E). In contrast, Wt1 expression was down-regulated in the cap mesenchyme but strongly up-regulated in more differentiated structures (Fig 2E). To further confirm these results, we also took advantage of *Osr1*^*GCE*^ knockin mice [29], which expressed an eGFP-Cre fusion protein from *Osr1* locus and examine the expression pattern of Osr1-GFP and endogenous Wt1 protein in *Osr1*^*GCE/+*^ embryos similarly (Fig 2F–2H). While TY1-Osr1 fusion protein displays a nuclear subcellular localization, whereas the Osr1-GFP is expressed in the cytoplasm, TY1-Osr1 and Osr1-GFP exhibited very similar expression pattern during kidney development (Fig 2C–2H).

To test the interaction of endogenous Osr1 and Wt1 *in vivo*, we dissected E16.5 kidneys from *Osr1*^*TY1/TY1*^ and wildtype embryos, respectively, and performed co-immunoprecipitation assays (Fig 2I). TY1-Osr1 was co-precipitated with endogenous Wt1 and Six2 (Fig 2I). Together, these results suggest that Osr1 and Wt1 interact in the nephrogenic mesenchyme to regulate kidney formation.

### Osr1 interacts genetically with Wt1 to regulate metanephric kidney development

**Fig 3:**
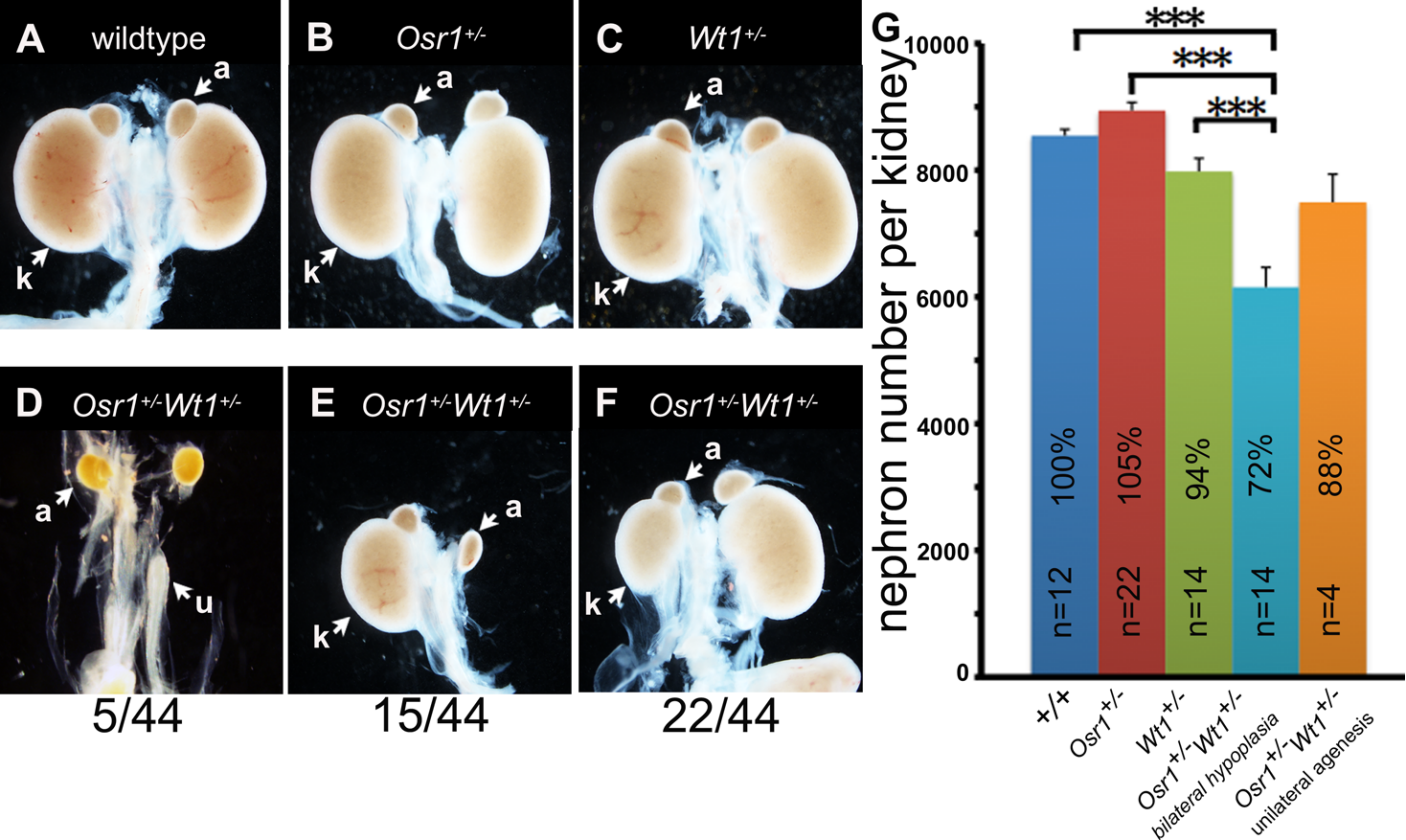
*Osr1*^*+/-*^*Wt1*^*+/-*^ mice exhibit kidney defects. (A-F) At P0, compared with wildtype (A), *Osr1*^*+/-*^ (B), and *Wt1*^***+/-***^ (C) mice, *Osr1*^*+/-*^*Wt1*^***+/-***^ mice exhibit kidney malformations, including bilateral renal agenesis (5/44) (D), unilateral renal agenesis (15/44) (E) and renal hypoplasia (22/44) (F). (G) Quantification of the number of nephrons per kidney in the wildtype (*+/+*), *Osr1*^*+/-*^, *Wt1*^*+/-*^, and *Osr1*^*+/-*^*Wt1*^*+/-*^ mice at P21. Error bar represents SEM. ***, p < 0.005.

We crossed *Osr1*^*+/-*^ mice with *Wt1*^*GFPCre/+*^ mice (abbreviated as *Wt1*^*+/-*^ mice in the rest of the article), in which a *GFP-Cre* fusion construct is inserted in the *Wt1* locus and disrupts the endogenous *Wt1* gene [40], and found that *Osr1*^*+/-*^*Wt1*^*+/-*^ double heterozygous pups exhibited variable kidney defects, including bilateral agenesis (5/44), unilateral agenesis (15/44) and bilateral hypoplasia (22/44) (Fig 3A–3F), while neither *Osr1*^*+/-*^ nor *Wt1*^*+/-*^ mice had any obvious kidney defects at birth. The surviving *Osr1*^*+/-*^*Wt1*^*+/-*^ mice with unilateral kidney agenesis had significant variation in kidney size and nephron number, but the mean nephron number per kidney was not statistically significantly different from the controls at P21 (Fig 3G). The number of nephrons per kidney of surviving *Osr1*^*+/-*^*Wt1*^*+/-*^ mice that had two kidneys was about 72% of that of their control littermates at P21, with the differences between these groups being highly significant (p < 0.005) (Fig 3G). Together, these results indicate that Osr1 and Wt1 act cooperatively to control metanephric kidney development.

### Osr1 interacts with Wt1 to regulate metanephric mesenchyme specification

**Fig 4:**
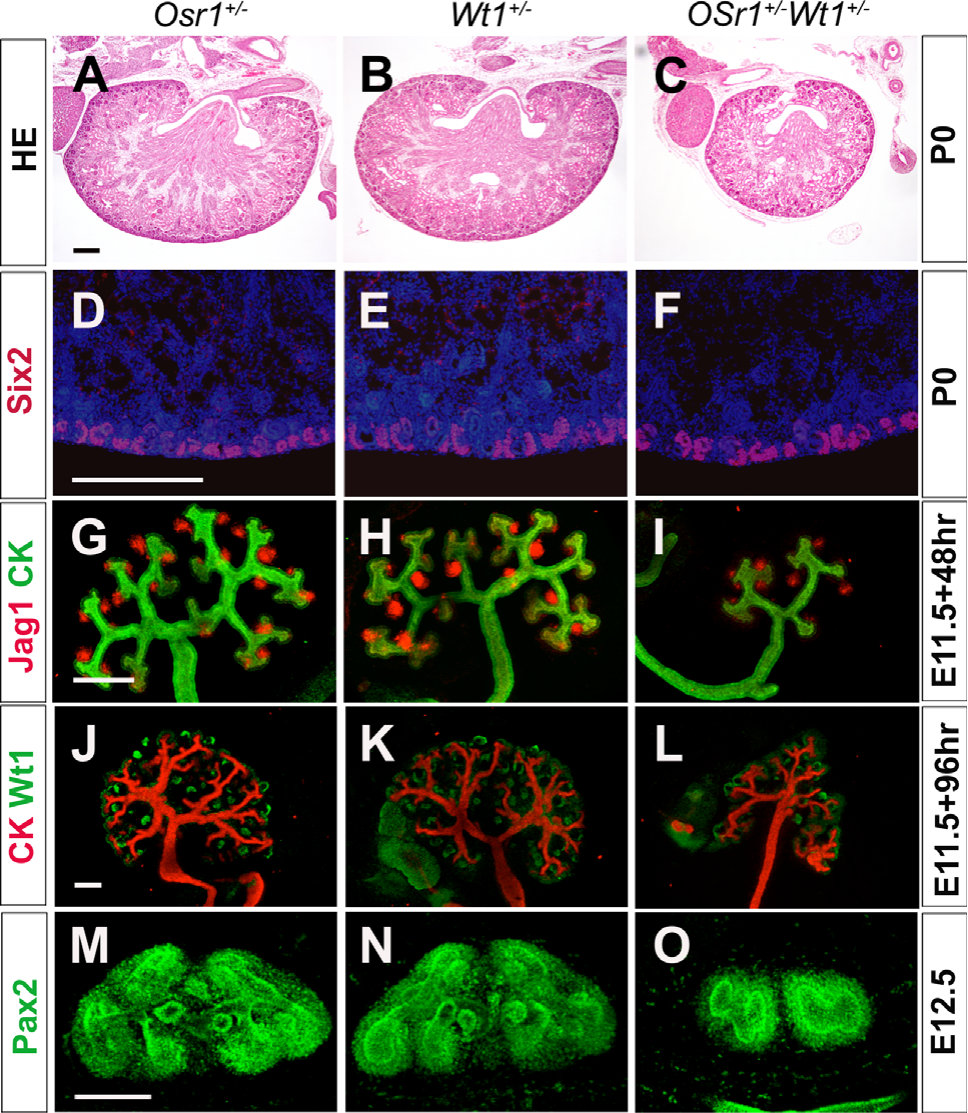
*Osr1*^*+/-*^*Wt1*^*+/-*^ embryos exhibit defects in ureteric bud branching. (A-C) HE staining of P0 kidney sections from *Osr1*^*+/-*^ (A), *Wt1*^*+/-*^ (B) and *Osr1*^*+/-*^*Wt1*^*+/-*^ (C) mice. (D-F) Immunofluorescent staining of Six2 (red) in P0 sections from *Osr1*^*+/-*^ (D), *Wt1*^*+/-*^ (E) and *Osr1*^*+/-*^*Wt1*^*+/-*^ (F) mice. Nuclear are co-staining with DAPI (Blue). (G-L) Whole mount immunofluorescence detection of Jag1 (red, G-I) or Wt1 (green, J-L) and pan-cytokeratin (CK, green in G-I, and red in J-L) in cultured *Osr1*^*+/-*^ (G, J), *Wt1*^*+/-*^ (H, K) and *Osr1*^*+/-*^*Wt1*^*+/-*^ (I, L) kidney explants. (M-O) Whole mount immunofluorescent detection of Pax2 (Green) in *Osr1*^*+/-*^ (M), *Wt1*^*+/-*^ (N) and *Osr1*^*+/-*^*Wt1*^*+/-*^ (O) kidney at E12.5. Scale bar, 200 μm.

We investigated whether the developmental abnormalities of the *Osr1*^*+/-*^*Wt1*^*+/-*^ mice could be due to impairment of maintenance of the nephron progenitor cells during metanephric kidney organogenesis. By P0, the kidneys in the *Osr1*^*+/-*^*Wt1*^*+/-*^ mice were obviously smaller then that in the *Osr1*^*+/-*^ and *Wt1*^*+/-*^ littermates (Fig 4A–4C). Histologically, however, the nephrogenic zone in the cortical region of kidney was present in the *Osr1*^*+/-*^*Wt1*^*+/-*^ mutants as well as in the *Osr1*^*+/-*^ and *Wt1*^*+/-*^ littermates (Fig 4A–4C). Immunofluorescent staining of the Six2 protein, a marker of nephron progenitor cells, on serial sections of the P0 kidneys showed the presence of Six2+ nephron progenitor cells in similar proportions in the *Osr1*^*+/-*^*Wt1*^*+/-*^ mutants as in the *Osr1*^*+/-*^ and *Wt*^*+/-*^ mice (Fig 4D–4F). This result indicates that the smaller than normal kidney size in the *Osr1*^*+/-*^*Wt1*^*+/-*^ mice is not due to premature differentiation of the nephron progenitor cells.

To investigate further the kidney developmental processes affected in the *Osr1*^*+/-*^*Wt1*^*+/-*^ embryos, we analyzed kidney morphogenesis using organ cultures. In 48 hours of organ culture, metanephric explants from E11.5 *Osr1*^*+/-*^ and *Wt1*^*+/-*^ embryos underwent 5 to 6 rounds of UB branching, with Jag1^+^ renal vesicles forming on the medullary side of each new ureteric bud branch (Fig 4G and 4H). In explants from *Osr1*^*+/-*^*Wt1*^*+/-*^ embryos, there were significantly fewer ureteric bud branches after 48 hours of culture (Fig 3I). After 96 hours of culture, *Osr1*^*+/-*^*Wt1*^*+/-*^ explants displayed fewer ureteric bud branches and obviously fewer Wt1-positive differentiating nephrons than in the *Osr1*^*+/-*^ or *Wt1*^*+/-*^ explants (Fig 4J–4L). We further examined UB branching *in vivo* by whole mount immunofluorescent staining for the Pax2 protein, which is expressed in both the UB and MM cells. At E12.5, compared with the kidneys in the *Osr1*^*+/-*^ and *Wt1*^*+/-*^ embryos, which had gone through 4 to 5 rounds of UB branching (Fig 4M and 4N), the kidneys of *Osr1*^*+/-*^*Wt1*^*+/-*^ mutant littermates showed apparently reduced UB branching (Fig 4O). These results indicate that ureteric bud growth and branching morphogenesis are impaired in *Osr1*^*+/-*^*Wt1*^*+/-*^ embryos compared with the *Osr1*^*+/-*^ and *Wt1*^*+/-*^ embryos.

**Fig 5:**
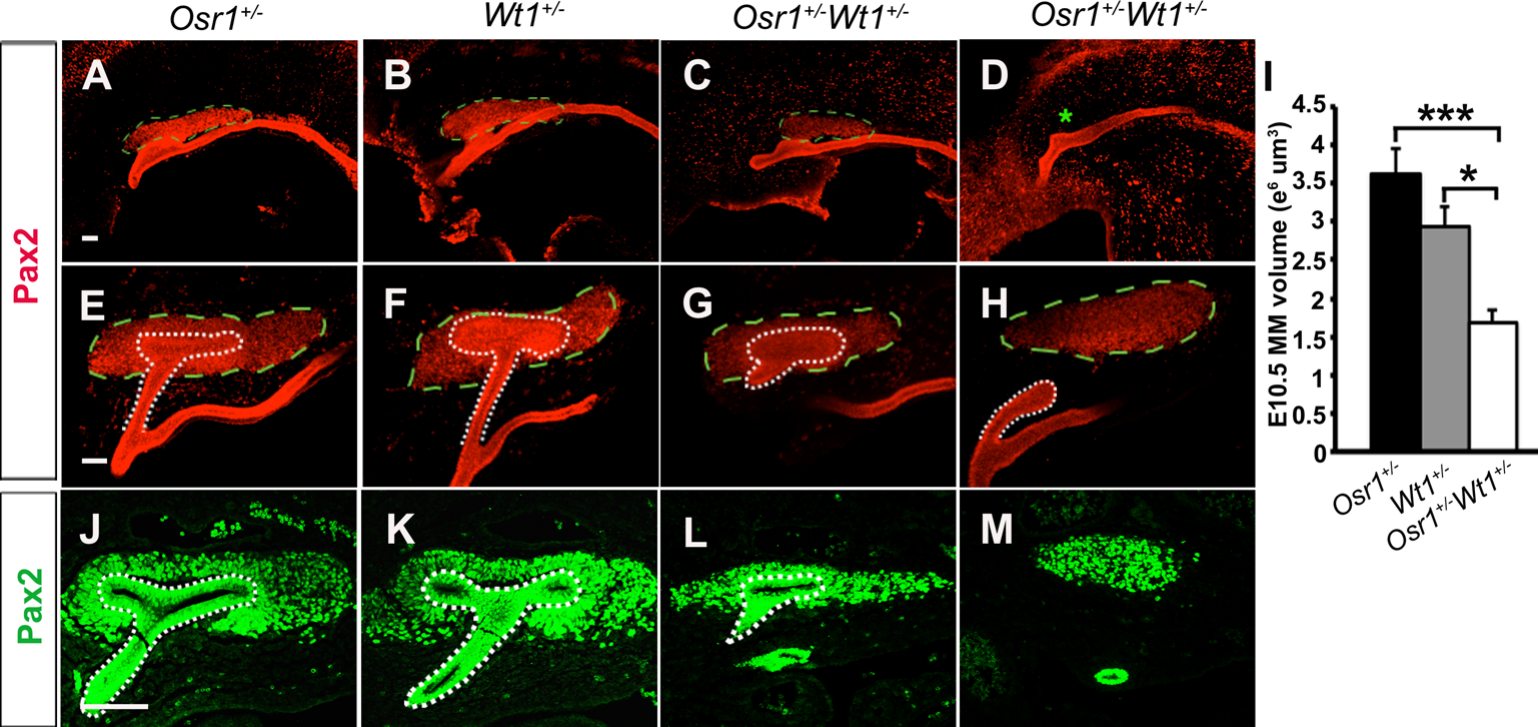
*Osr1*^*+/-*^*Wt1*^*+/-*^ embryos exhibit defects in metanephric mesenchyme. (A-H) Whole mount immunofluorescent staining for Pax2 (red) at E10.5 (A-D) and E11.5 (E-H). The white dot line outlines the ureteric bud epithelium. The green dashed line outlines the MM domain. Asterisk in D indicates that no condensed Pax2+ MM domain was detected. (I) Quantification of MM volume at E10.5 (n = 10). The MM volume does not include UB or duct epithelium. Error bar represent SEM. *, p < 0.05; ***, p < 0.005. (J-M) Immunofluorescent staining for Pax2 (green) in E11.5 sections. Scale bar, 100 μm.

To define the onset and progression of kidney developmental defects in the *Osr1*^*+/-*^*Wt1*^*+/-*^ mice, we compared the formation of MM and UB in the *Osr1*^*+/-*^*Wt1*^*+/-*^ embryos with that of the *Osr1*^*+/-*^ and *Wt1*^*+/-*^ littermates at E10.5 and E11.5, respectively. By E10.5, the MM is clearly demarcated by Pax2 protein expression in the control embryos. Whole mount immunofluorescent detection of Pax2 protein showed that the domain of MM is significantly smaller in the *Osr1*^*+/-*^*Wt1*^*+/-*^ embryos than that in the *Osr1*^*+/-*^ and *Wt1*^*+/-*^ embryos at E10.5 (Fig 5A–5C and 5I). In 2 out of 12 *Osr1*^*+/-*^*Wt1*^*+/-*^ embryos, no condensed Pax2+ MM domain was detected at E10.5 (Fig 5D). We also performed whole mount immunofluorescent staining for Six2, a more specific marker for the MM cells, and found that the Six2+ MM domain was apparently smaller in the *Osr1*^*+/-*^*Wt1*^*+/-*^ embryos than that in the *Osr1*^*+/-*^ and *Wt1*^*+/-*^ embryos at E10.5 (S1 Fig). By E11.5, the ureteric bud had undergone the first round of branching morphogenesis, forming a “T” shape and the MM cells were condensed around the UB tips, in wildtype as well as in the *Osr1*^*+/-*^ and *Wt1*^*+/-*^ embryos (Fig 5E, 5F, 5J and 5K). In E11.5 *Osr1*^*+/-*^*Wt1*^*+/-*^ embryos, however, the UBs were smaller and fewer MM cells surrounded the UB tips (Fig 5G and 5L). In 2 out of 10 *Osr1*^*+/-*^*Wt1*^*+/-*^ embryos, the UB failed to invade into the MM by E11.5 (Fig 5H and 5M). In 1 out of 10 *Osr1*^*+/-*^*Wt1*^*+/-*^ embryos, the UB reached the MM but had not branched by E11.5.

**Fig 6:**
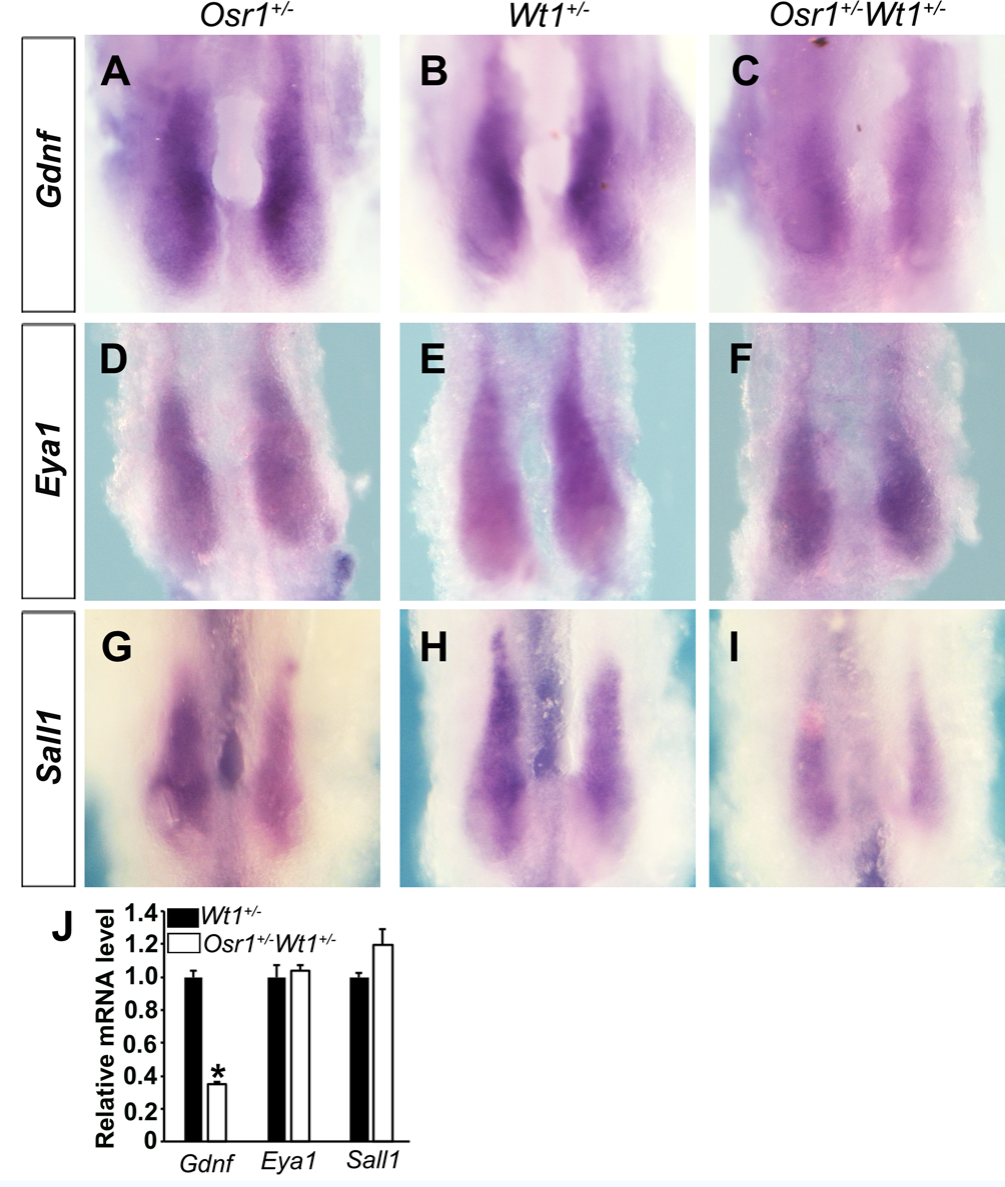
Molecular marker expression in the metanephric mesenchyme in *Osr1*^*+/-*^*Wt1*^*+/-*^ embryos and littermates. Whole mount *in situ* hybridization detection of *Gdnf* (A-C), *Eya1* (D-F), and *Sall1* (G-I) mRNA expression in E10.5 embryos. Scale bar, 200 μm. (J) Real-time RT-PCR analysis of the levels of expression of *Gdnf*, *Eya1*, and *Sall1* mRNAs in Wt1-GFP+ cells from E9.5 *Osr1*^*+/-*^*Wt1*^*+/-*^ embryos and *Wt1*^*+/-*^ littermate. *, p < 0.05.

A number of genes expressed in the MM, including *Gdnf*, *Eya1*, *Sall1*, and *Pax2* are each essential for UB induction and early kidney morphogenesis [7–9, 16, 17, 41, 42]. We analyzed the expression patterns of *Gdnf*, *Eya1* and *Sall1* in *Osr1*^*+/-*^*Wt1*^*+/-*^ embryos and their littermates at E10.5 by whole mount *in situ* hybridization and found that the expression domain of *Gdnf*, *Eya1* and *Sall1* were reduced, and the levels of *Gdnf* were decreased in the nephrogenic mesenchyme in *Osr1*^*+/-*^*Wt1*^*+/-*^ embryos compared with the *Osr1*^*+/-*^ and *Wt1*^*+/-*^ embryos (Fig 6A–6I). To further quantify the levels of expression of these marker genes in the nephrogenic mesenchyme, we took advantage of the GFP expression from the *Wt1*^*GFPCre*^ allele [40] to isolate the Wt1-GFP+ cells from *Osr1*^*+/-*^*Wt1*^*+/-*^ and *Wt1*^*+/-*^ embryos, respectively, and performed quantitative real-time RT-PCR assays. We found that the level of *Gdnf* mRNAs was significantly reduced, but the levels of *Eya1* and *Sall1* were not significantly changed, in *Osr1*^*+/-*^*Wt1*^*+/-*^ embryos compared with *Wt1*^*+/-*^ embryos at E9.5 (Fig 6J). These results indicate a critical role for the synergistic actions of Osr1 and Wt1 in the specification of the MM during early nephrogenesis.

### The *Osr1*^*+/-*^*Wt1*^*+/-*^ embryos had no aberrant apoptosis or cell proliferation during early nephrogenesis

**Fig 7:**
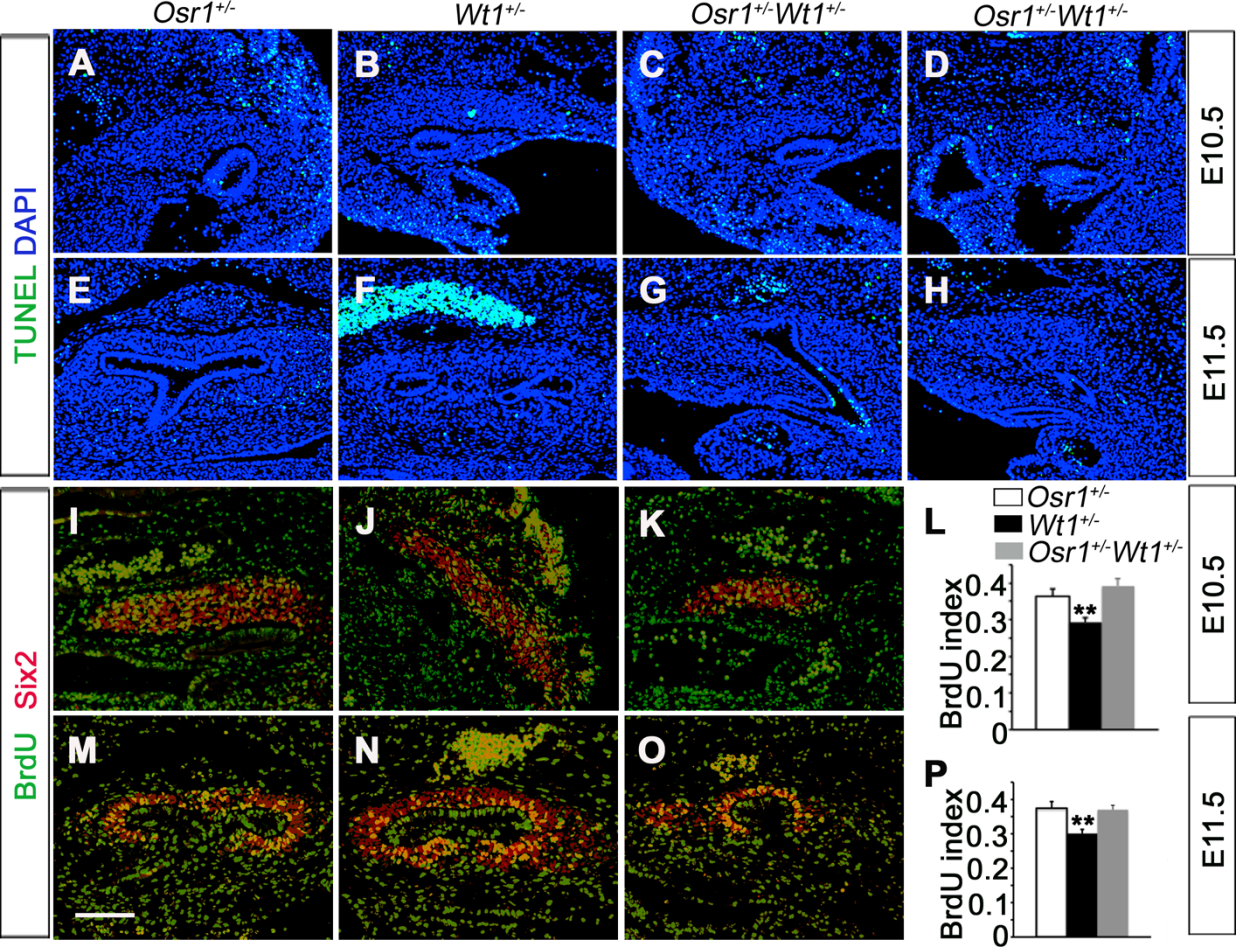
Analysis of cell apoptosis and proliferation during early kidney development in *Osr1*^*+/-*^*Wt1*^*+/-*^ embryos and littermates. (A-H) Cell apoptosis is analyzed by TUNEL (green) and counterstained with DAPI (blue). TUNEL assay detected no obvious change in cell apoptosis in *Osr1*^*+/-*^*Wt1*^*+/-*^ metanephric mesenchyme (C, D, G, H) compared with *Osr1*^*+/-*^ (A, E) and *Wt1*^*+/-*^ (B, F) metanephric mesenchyme at E10.5 (A-D) and E11.5 (E-H). (I-P) Analysis of cell proliferation by BrdU incorporation at E10.5 (I-L) (n = 18) and E11.5 (n = 7) (M-P) embryos. BrdU index is calculated by the ratio of BrdU-positive cells (green) versus Six2-positive cells (red). Scale bar, 100 μm.

Both *Osr1*^*-/-*^ and *Wt1*^*-/-*^ mutant mouse embryos had aberrant apoptosis of the nephrogenic mesenchyme at the early stages of kidney development [27, 28, 33, 43]. Therefore, we examined cell proliferation and apoptosis in the *Osr1*^*+/-*^*Wt1*^*+/-*^ embryos and their littermates at E10.5 and E11.5 (Fig 7). Very few apoptotic cells and no significant differences in the proportion of cells undergoing apoptosis were detected in the metanephric mesenchyme in the *Osr1*^*+/-*^, *Wt1*^*+/-*^, and *Osr1*^*+/-*^*Wt1*^*+/-*^ embryos at both stages (Fig 7A–7H). To determine if there was any difference in the proliferation of MM cells, we used immunofluorescent staining of Six2 to mark the MM cell nuclei and compared the percentage of BrdU-labeled MM cells in the *Osr1*^*+/-*^, *Wt1*^*+/-*^, and *Osr1*^*+/-*^*Wt1*^*+/-*^ embryos at E10.5 and E11.5. We didn’t detect a significant difference in the cell proliferation index in the MM of *Osr1*^*+/-*^*Wt1*^*+/-*^ embryos and the *Osr1*^*+/-*^ littermates at either stage (Fig 7I–7P). These results indicate that the renal agenesis or hypoplasia in the *Osr1*^*+/-*^*Wt1*^*+/-*^ mutant embryos resulted primarily from an impairment of MM specification rather than from increased MM apoptosis or reduced MM cell proliferation.

## Discussion

In humans, the number of nephrons per kidney varies greatly, ranging from 200,000 to >2.5 million, among individuals [1]. Renal hypoplasia, with abnormally low number of nephrons, underlies more than one third of pediatric chronic or end-stage kidney diseases and is a significant risk factor for hypertension in adulthood [1, 2]. Whereas the mechanisms that determine the number of nephrons are incompletely understood, genetic studies in mice in recent years have provided significant insights into the molecular regulation of nephrogenesis and the developmental basis of renal hypoplasia [44]. Since *Osr1* is expressed in nephrogenic progenitor cells throughout kidney development and since mice lacking *Osr1* function exhibit complete kidney agenesis [27, 28], it was hypothesized that genetic variation in *OSR1* might contribute to the variation in nephron number in humans [32]. Indeed, a single nucleotide polymorphism, rs12329305 (C/T), residing in an exonic splice enhancer in the human *OSR1* gene, has been associated with reduction in newborn kidney size and function [31]. However, how Osr1 functions to regulate nephron number is not known. Previous studies showed that nephrogenic mesenchyme underwent aberrant apoptosis prior to UB induction in the *Osr1*^*-/-*^ embryos [27, 28]. Recently, we demonstrated that Osr1 interacts with Six2 to maintain nephron progenitor cells in the CM against Wnt/β-catenin driven nephron differentiation [30]. In this study we found that, although the *Osr1*^*+/-*^ and *Wt1*^*+/-*^ mice did not have obvious kidney developmental defects, the majority of *Osr1*^+/-^*Wt1*^*+/-*^ had kidney agenesis or hypoplasia. Our data demonstrate that, in addition to the previously determined roles in nephrogenic mesenchyme survival and CM maintenance, Osr1 interacts with Wt1 to regulate specification of the MM.

The *Wt1* gene encodes a zinc finger protein that acts both as a transcription factor and RNA-binding protein [45–47]. *Wt1* expression is activated in the nephrogenic mesenchyme as early as E9.0 during mouse embryogenesis and *Wt1*^*-/-*^ mouse embryos showed complete lack of metanephric kidneys [33]. However, in contrast to the lack of morphologically distinguishable MM in the E10.5 *Osr1*^*-/-*^ embryos [27, 28], *Wt1*^*-/-*^ embryos not only had morphologically distinguishable MM by E10.5 but also expressed the MM molecular markers *Pax2* and *Gdnf* mRNAs prior to aberrant apoptosis at E12 [33, 48]. In this study, we found that *Osr1*^*+/-*^*Wt1*^*+/-*^ double heterozygous embryos had reduced MM domain to about half of that in the control littermates at E10.5. The expression of *Gdnf* in the MM is also reduced in the *Osr1*^*+/-*^*Wt1*^*+/-*^ embryos in comparison with the *Osr1*^*+/-*^ and *Wt1*^*+/-*^ littermates. Together with our data demonstrating overlapping expression and protein-protein interaction between Osr1 and Wt1, these results indicate that both Osr1 and Wt1 play critical roles in MM specification.

We previously showed that *Osr1*^*-/-*^ embryos failed to activate expression of MM markers, including *Eya1*, *Pax2*, and *Six2*, at E10.5 [28]. Since the *Osr1*^*-/-*^ embryos exhibit aberrant apoptosis of the nephrogenic mesenchyme starting at E9.5 [27], however, it had not been clear whether Osr1 plays a direct role in MM formation and whether the lack of MM in *Osr1*^*-/-*^ embryos was secondary to the requirement of Osr1 for nephrogenic mesenchyme survival. On the other hand, the *Wt1*^*-/-*^ mutant embryos showed morphologically distinguishable MM at E10.5 and E11.5 but the *Wt1*^*-/-*^ MM did not respond to wildtype UB while UB outgrowth was induced from *Wt1*^*-/-*^ mutant nephric duct by wildtype MM in recombinant explant cultures [48], indicating that Wt1 is required cell autonomously in the MM for production and/or processing of UB-inducing signals and for competence to respond to UB-derived nephrogenic signals. In this study, we found that the *Osr1*^*+/-*^*Wt1*^*+/-*^ embryos had reduced MM in the absence of any detectable defects in cell proliferation or apoptosis. These results clearly demonstrate that synergistic interactions of Osr1 and Wt1 play critical roles in MM formation and function.

We recently demonstrated that tissue-specific inactivation of *Osr1* in the cap mesenchyme resulted in premature differentiation and depletion of nephron progenitor cells [30]. In this study, we found that the *Osr1*^*+/-*^*Wt1*^*+/-*^ mutant embryos had normal maintenance of the CM nephron progenitor cells even though the kidneys were reduced in size. A recent study showed that genetically ablating 40% of the nephron progenitor cells (cap mesenchymal cells) caused decreased ureteric bud branching with normal CM condensation around the UB tips and resulted in hypoplastic kidneys [49]. Thus, the number of nephron progenitor cells at the beginning of metanephric kidney organogenesis is an important determinant of nephron endowment [49]. Our results indicate that impairment in MM specification is one of the mechanisms underlying renal hypoplasia.

Zhang et al. (2011) showed that kidney volume was more significantly reduced in infants bearing both the *OSR1*^*rs12329305(T)*^ allele and the *RET*^*rs1800860(A)*^ allele, suggesting that the effects of impaired *OSR1* function on nephrogenesis was additive with other regulators of UB branching [31, 32]. We found that the *Osr1*^*+/-*^*Wt1*^*+/-*^ mouse embryos display reduced MM volume and significantly decreased *Gdnf* expression. Since Gdnf is the major signal and acts primarily through the Ret receptor for UB induction [6–10], the reduced *Gdnf* expression likely contributes to the reduction in UB branching in the *Osr1*^*+/-*^*Wt1*^*+/-*^ mouse embryos and is likely part of the mechanism underlying renal hypoplasia in infants heterozygous for the *OSR1*^*rs12329305(T)*^ allele. The synergistic interaction of Osr1 and Wt1 in the regulation of MM specification, together with the finding that about 6% of the Caucasian population carries the functionally impaired *OSR1*^*rs12329305(T)*^ allele [31, 32], suggest that *OSR1* has a significant contribution to renal hypoplasia and other renal conditions in humans. Further studies of the molecular mechanisms involving Osr1 in kidney development, in particular identification of direct downstream target genes and its interaction with other transcription factors and molecular pathways, will significantly improve the understanding of renal disease pathogenesis.

## Materials and Methods

### Mouse strains

This study was performed in strict accordance with the recommendations in the Guide for the Care and Use of Laboratory Animals by the National Institutes of Health. The animal use protocol was approved by the Institutional Animal Care and Use Committee of Cincinnati Children’s Hospital Medical Center (Permit Number IACUC2013-0036). The mice were housed in standard microisolater cages with ventilation, standard mouse food, and automated water supply. Breeder male mice are housed individually and adult female mice are housed at 4 mice/cage. To harvest mouse embryos for the experimental studies, timed pregnant female mice were euthanized by asphyxiation with carbon dioxide gas generated from a pressurized cylinder followed by cervical dislocation in accordance with the Panel on Euthanasia of the American Veterinary Medical Association. Embryos were fixed in 4% paraformaldehyde immediately upon dissection. All methods and procedures are reviewed for humaneness by the Institutional Animal Care and Use Committee. When necessary, consultation and training in the application of analgestic and anesthetics for laboratory animals are provided through an annual training program supplemented by individualized training in consultation with the veterinarian.

The *Osr1*^*+/-*^ (*Osr1*^*tm1Jian*^), *Osr1*^*GCE/+*^ and *Wt1*^*+/-*^ (*Wt1*^*GFPcre/+*^) mice have been described previously [27, 29, 40]. The *Osr1*^*+/-*^ (*Osr1*^*tm1Jian*^) and *Osr1*^*GCE/+*^ were maintained by crossing to C57BL/6J mice. *Osr1*^*+/-*^*Wt1*^*+/-*^ male mice were crossed with CD1 (Charles river) female mice to generate *Osr1*^*+/-*^, *Wt1*^*+/-*^ single heterozygous and *Osr1*^*+/-*^*Wt1*^*+/-*^ double heterozygous mice. Noon of the day a vaginal plug was identified was designated as embryonic day (E) 0.5.

### Generation of mice carrying the *Osr1*^*TY1*^ allele using CRISPR/Cas9-mediated genome editing

A guide RNA (gRNA) targeting sequence near the translation start site in Exon2 of the *Osr1* gene (gRNA target sequence 5’- GCGGTTCCCAGCGACAGAAA-3’) was selected using the CRISPR Design Tool (http://crispr.mit.edu). Guide RNAs were synthesized *in vitro* and co-injected with a synthesized single-stranded oligonucleotide donor (5’- ttgggtctgtccccaccctcttcctgtctttgcagatatttgaaatcattgcggttcccagcgacagCCATGGAGGTGCACACCAACCAGGACCCCCTGGACGCCGAAGTCCATACAAATCAGGATCCTCTGGATGCCGCAatgGgcagcaaaaccttgccagcaccggtacccattcacccatccctgcagctt -3’) as template for homology-directed repair to insert the 2xTY1 epitope tag immediately 5’ to the translation start site, and humanized *Cas9* mRNAs into zygotes from B6D2F1 (C57BL/6 X DBA2) mice to generate gene-targeted mice in the CCHMC Transgenic Animal and Genome Editing Facility (the concentrations for the gRNA, oligonucleotide donor template, and *Cas9* mRNAs were 50 ng/μl, 100 ng/μl, and 100 ng/μl, respectively). Transgenic founder mice were identified by PCR genotyping of genomic DNA isolated from tail biopsies using the primers Osr1nF 5’-GGCAGTAGGTTCATGGGTGG-3’ and Osr1nR 5’-CATACAGGTTGGGCAGGTGG-3’, which produces PCR products of 311 bp and 377 bp, respectively from the wildtype and *Osr1*^*TY1*^ alleles. Out of 12 G0 founder mice, 4 carried the in-frame *2xTY1* insertion. All 4 positive G0 mice were bred to C57BL/6J inbred mice to test for germline transmission and 3 (one male and 2 female G0 founders) transmitted the correct in-frame *2xTY1* tag insertion. Genotypically verified G1 *Osr1*^*TY1/+*^ hemizygous mice were intercrossed to generate *Osr1*^*TY1/TY1*^ homozygotes.

### TUNEL and cell proliferation assays

Apoptotic cells were detected on 7μm paraffin sections using DeadEnd^TM^ Fluoro-metric TUNEL System (Promega, G3250) according to manufacturer’s instructions. Six kidney sections from each of two embryos of each genotype at each developmental stage were analyzed.

To determine proliferative activity of metanephric mesenchyme, timed-mated pregnant mice were injected intraperitoneally with 10 μl/g BrdU (5 mg/ml stock) (Sigma-Aldrich, B5002). Embryos were harvested 1 hour after injection. The BrdU-labeling index was defined as the number of BrdU-positive nuclei relative to Six2-positive nuclei, which was detected by immunostaining.

### Kidney explant culture

The metanephric rudiments were dissected from E11.5 mouse embryos and positioned on top of a culture plate insert (1.0 μm pore size, BD Falcon, 353102) within an individual well of a 6-well tissue culture plate and cultured in DMEM/F12 media plus 10% fetal bovine serum (Invitrogen). The explant cultures were maintained at 37°C at an atmosphere of 5% CO2 and 100% humidity.

### Nephron Number Quantification, Histology, and Immunofluorescent staining

The number of nephrons per kidney was measured using a previously established protocol [50].

For histological analysis, embryos were dissected at desired stages from timed pregnant mice, fixed in 4% paraformaldehyde (PFA), dehydrated through an ethanol series, embedded in paraffin, sectioned at 7μm thickness, and stained with hematoxylin and eosin.

Immunofluorescent staining of paraffin sections was performed following standard protocols. Antibodies and reagents used are: rabbit anti-Six2 (ProteinTech, 11562-1-AP), mouse anti-Pan cytokeratin (Sigma), rabbit anti-Jagged1 (Santa Cruz, sc-8303), rabbit anti-Wt1 (Santa Cruz, sc-192), rabbit anti-Pax2 (Invitrogen, 71–6000), chicken anti-GFP (AVES labs, GFP-1010).

For whole mount immunostaining staining, E10.5 lower trunk or E11.5 urogenital regions were dissected and fixed in 1% PFA at 4°C over night. After fixation, embryos were washed, and transferred to 100% methanol. The embryos were bleached in a solution of methanol/H2O2/DMSO (4:1:1) for 2 hours at room temperature. After rehydration, embryos were blocked with 2.5% goat serum and 5% BSA in PBST for 2 hours, incubated in primary antibody diluted in blocking buffer overnight at 4°C. Embryos were washed, and then incubated with secondary antibody overnight at 4°C. Specimens were examined and photographed using a Nikon inverted confocal microscope. The volume of MM was analyzed by Imaris software after confocal imaging.

### *In situ* hybridization

Whole mount *in situ* hybridization was performed as previously described. At least three embryos of each genotype were hybridized to each probe and only probes that detected consistent patterns of expression in all samples were considered as valid results [26].

### Co-immunoprecipitation

The *Osr1* coding sequence was subcloned into the pCS2 vector to express Osr1 with Myc-epitope tag. *Osr1*, *Wt1*, *Lhx1* and *Six2* coding sequences were subcloned into pCMV7.1 (Sigma) vectors with a Flag-tag. Pax2 coding sequence was subcloned into pcDNA3 (invitrogen). Flag-Six2, Flag-Lhx1 and Myc-Pax2 plasmids were kindly provided by Dr. Joo-Seop Park (Cincinnati Children’s Hospital, Medical Center).

For immunoprecipitation assays, HEK293T cells were co-transfected with plasmids as indicated. After transfection, cells were cultured in DMEM supplemented with 10% fetal bovine serum for 48 hours. The cells were lysed in RIPA buffer containing proteinase inhibitors (Santa Cruz, SC-24948). Whole cell lysate was incubated with anti-c-Myc antibody conjugated to protein-G agarose beads (4A6, Millipore, 16–219), or anti-Flag (M2, sigma, F1804) coupled with protein-G dynabeads (Thermo Fisher scientific, 10003D) and rotated at 4°C over night. The beads were washed five times with RIPA buffer. Western blot was performed using anti-Flag (M2, Sigma F3165), anti-c-Myc (4A6, Millipore 05–724) antibodies. E16.5 kidney samples from *Osr1*^*TY1/TY1*^ or wildtype embryos were harvested and lysed in lysis buffer (50 mM HEPES [pH 7.5], 140 mM NaCl, 1 mM EDTA, 0.5% NP-40, 0.25% TX-100, and 1x complete mini protease inhibitor [Roche, 04693159001]). Lysates were incubated with anti-TY1 antibody (Diagenode, C15200054) coupled dynabeads at 4 C for overnight. The beads were washed five times with lysis buffer. The western blot was performed with anti-Six2 anti-body (Proteintech, 11562-1-AP), anti-Flag antibody (M2, Sigma, F1804), or anti-Wt1 antibody (Santa Cruz, SC-192).

### FACS sorting and real-time RT-PCR

The trunk tissues of E9.5 embryos from wildtype females crossed with *Osr1*^*+/-*^*Wt1*^*+/-*^ double heterozygous males were manually microdissected and digested with trypsin-EDTA (Invitrogen) at 37°C for 4 minutes. After inactivation of trypsin with DMEM containing 10% FBS, cells were dissociated by pipetting. The dissociated cells were resuspended in PBS with 2% FBS and 10 mM EDTA, and filtered through a 40 μm nylon cell strainer (BD Falcon, 352340). GFP+ cells were isolated using BD FACSAria II. FACS-isolated GFP+ cells from *Osr1*^*+/-*^*Wt1*^*+/-*^ double heterozygous embryos and *Wt1*^*+/-*^ heterozygous littermates were used for RNA extraction. First-strand cDNAs were synthesized by the Gene Expression Core at Cincinnati Children’s Hospital Medical Center. Real-time PCR was performed using a Bio-Rad CFX96 Real-Time System using conditions recommended by the manufacturer. Each reaction was performed in triplicates. The quantity of each mRNA was first determined using a standard curve method and normalized to the internal control (β-Actin).

### Statistical analysis

All results were presented as mean ± SEM. All statistical analyses were done using Excel software. Two-tailed Student’s t tests were used for comparisons between two groups. P value less than 0.05 was considered significant.

## Supporting Information

S1 Fig *Osr1*^*+/-*^*Wt1*^*+/-*^ embryos exhibit defects in Six2-positive metanephric mesenchyme. (A-C) Whole mount immunofluorescent staining for Six2 protein (red) in E10.5 *Osr1*^*+/-*^ (A), *Wt*^*+/-*^ (B), and *Osr1*^*+/-*^*Wt1*^*+/-*^ (C) embryos. The embryos were counterstained with DAPI (Blue). The white dotted line outlines the metanephric mesenchyme. Scale bar, 100 μm.
(TIF)

## References

[1] Bertram JF, Douglas-Denton RN, Diouf B, Hughson MD, Hoy WE. Human nephron number: implications for health and disease. Pediatr Nephrol. 2011;26(9):1529–33. Epub 2011/05/24. 10.1007/s00467-011-1843-8 21604189

[2] Keller G, Zimmer G, Mall G, Ritz E, Amann K. Nephron number in patients with primary hypertension. N Engl J Med. 2003;348(2):101–8. Epub 2003/01/10. 12519920 10.1056/NEJMoa020549

[3] Cain JE, Di Giovanni V, Smeeton J, Rosenblum ND. Genetics of renal hypoplasia: insights into the mechanisms controlling nephron endowment. Pediatr Res. 2010;68(2):91–8. 10.1203/00006450-201011001-00175 20421843

[4] Little MH, Bertram JF. Is there such a thing as a renal stem cell? J Am Soc Nephrol. 2009;20(10):2112–7. Epub 2009/08/29. 10.1681/ASN.2009010066 19713310

[5] Little MH, McMahon AP. Mammalian kidney development: principles, progress, and projections. Cold Spring Harb Perspect Biol. 2012;4(5). Epub 2012/05/03. 10.1101/cshperspect.a008300 PMC3331696 22550230

[6] Durbec P, Marcos-Gutierrez CV, Kilkenny C, Grigoriou M, Wartiowaara K, Suvanto P, et al GDNF signalling through the Ret receptor tyrosine kinase. Nature. 1996;381(6585):789–93. Epub 1996/06/27. 8657282 10.1038/381789a0

[7] Moore MW, Klein RD, Farinas I, Sauer H, Armanini M, Phillips H, et al Renal and neuronal abnormalities in mice lacking GDNF. Nature. 1996;382(6586):76–9. Epub 1996/07/04. 8657308 10.1038/382076a0

[8] Pichel JG, Shen L, Sheng HZ, Granholm AC, Drago J, Grinberg A, et al Defects in enteric innervation and kidney development in mice lacking GDNF. Nature. 1996;382(6586):73–6. Epub 1996/07/04. 8657307 10.1038/382073a0

[9] Sanchez MP, Silos-Santiago I, Frisen J, He B, Lira SA, Barbacid M. Renal agenesis and the absence of enteric neurons in mice lacking GDNF. Nature. 1996;382(6586):70–3. Epub 1996/07/04. 8657306 10.1038/382070a0

[10] Vega QC, Worby CA, Lechner MS, Dixon JE, Dressler GR. Glial cell line-derived neurotrophic factor activates the receptor tyrosine kinase RET and promotes kidney morphogenesis. Proc Natl Acad Sci U S A. 1996;93(20):10657–61. Epub 1996/10/01. 8855235 10.1073/pnas.93.20.10657 PMC38210

[11] Schuchardt A, D'Agati V, Pachnis V, Costantini F. Renal agenesis and hypodysplasia in ret-k- mutant mice result from defects in ureteric bud development. Development. 1996;122(6):1919–29. Epub 1996/06/01. 8674430 10.1242/dev.122.6.1919

[12] Jijiwa M, Fukuda T, Kawai K, Nakamura A, Kurokawa K, Murakumo Y, et al A targeting mutation of tyrosine 1062 in Ret causes a marked decrease of enteric neurons and renal hypoplasia. Mol Cell Biol. 2004;24(18):8026–36. 15340065 10.1128/MCB.24.18.8026-8036.2004 PMC515068

[13] Cullen-McEwen LA, Drago J, Bertram JF. Nephron endowment in glial cell line-derived neurotrophic factor (GDNF) heterozygous mice. Kidney Int. 2001;60(1):31–6. 11422733 10.1046/j.1523-1755.2001.00767.x

[14] Cullen-McEwen LA, Kett MM, Dowling J, Anderson WP, Bertram JF. Nephron number, renal function, and arterial pressure in aged GDNF heterozygous mice. Hypertension. 2003;41(2):335–40. 12574104 10.1161/01.hyp.0000050961.70182.56

[15] Torres M, Gomez-Pardo E, Dressler GR, Gruss P. Pax-2 controls multiple steps of urogenital development. Development. 1995;121(12):4057–65. Epub 1995/12/01. 8575306 10.1242/dev.121.12.4057

[16] Xu PX, Adams J, Peters H, Brown MC, Heaney S, Maas R. Eya1-deficient mice lack ears and kidneys and show abnormal apoptosis of organ primordia. Nat Genet. 1999;23(1):113–7. 10471511 10.1038/12722

[17] Xu PX, Zheng W, Huang L, Maire P, Laclef C, Silvius D. Six1 is required for the early organogenesis of mammalian kidney. Development. 2003;130(14):3085–94. Epub 2003/06/05. 12783782 10.1242/dev.00536 PMC3872112

[18] Chung GW, Edwards AO, Schimmenti LA, Manligas GS, Zhang YH, Ritter R, 3rd. Renal-coloboma syndrome: report of a novel PAX2 gene mutation. American journal of ophthalmology. 2001;132(6):910–4. 11730657 10.1016/s0002-9394(01)01231-4

[19] Ford B, Rupps R, Lirenman D, Van Allen MI, Farquharson D, Lyons C, et al Renal-coloboma syndrome: prenatal detection and clinical spectrum in a large family. Am J Med Genet. 2001;99(2):137–41. 11241473 10.1002/1096-8628(2000)9999:999<00::aid-ajmg1143>3.0.co;2-f

[20] Ruf RG, Xu PX, Silvius D, Otto EA, Beekmann F, Muerb UT, et al SIX1 mutations cause branchio-oto-renal syndrome by disruption of EYA1-SIX1-DNA complexes. Proc Natl Acad Sci U S A. 2004;101(21):8090–5. 15141091 10.1073/pnas.0308475101 PMC419562

[21] Morisada N, Rendtorff ND, Nozu K, Morishita T, Miyakawa T, Matsumoto T, et al Branchio-oto-renal syndrome caused by partial EYA1 deletion due to LINE-1 insertion. Pediatr Nephrol. 2010;25(7):1343–8. 10.1007/s00467-010-1445-x 20130917

[22] Weber S, Taylor JC, Winyard P, Baker KF, Sullivan-Brown J, Schild R, et al SIX2 and BMP4 mutations associate with anomalous kidney development. J Am Soc Nephrol. 2008;19(5):891–903. Epub 2008/02/29. 10.1681/ASN.2006111282 18305125 PMC2386720

[23] Porteous S, Torban E, Cho NP, Cunliffe H, Chua L, McNoe L, et al Primary renal hypoplasia in humans and mice with PAX2 mutations: evidence of increased apoptosis in fetal kidneys of Pax2(1Neu) +/- mutant mice. Hum Mol Genet. 2000;9(1):1–11. 10587573 10.1093/hmg/9.1.1

[24] Self M, Lagutin OV, Bowling B, Hendrix J, Cai Y, Dressler GR, et al Six2 is required for suppression of nephrogenesis and progenitor renewal in the developing kidney. EMBO J. 2006;25(21):5214–28. Epub 2006/10/13. 17036046 10.1038/sj.emboj.7601381 PMC1630416

[25] So PL, Danielian PS. Cloning and expression analysis of a mouse gene related to Drosophila odd-skipped. Mech Dev. 1999;84(1–2):157–60. Epub 1999/09/03. 10473132 10.1016/s0925-4773(99)00058-1

[26] Lan Y, Kingsley PD, Cho ES, Jiang R. Osr2, a new mouse gene related to Drosophila odd-skipped, exhibits dynamic expression patterns during craniofacial, limb, and kidney development. Mechanisms of development. 2001;107(1–2):175–9. 11520675 10.1016/s0925-4773(01)00457-9

[27] Wang Q, Lan Y, Cho ES, Maltby KM, Jiang R. Odd-skipped related 1 (Odd 1) is an essential regulator of heart and urogenital development. Dev Biol. 2005;288(2):582–94. Epub 2005/10/15. 16223478 10.1016/j.ydbio.2005.09.024 PMC3869089

[28] James RG, Kamei CN, Wang Q, Jiang R, Schultheiss TM. Odd-skipped related 1 is required for development of the metanephric kidney and regulates formation and differentiation of kidney precursor cells. Development. 2006;133(15):2995–3004. Epub 2006/06/23. 16790474 10.1242/dev.02442

[29] Mugford JW, Sipila P, McMahon JA, McMahon AP. Osr1 expression demarcates a multi-potent population of intermediate mesoderm that undergoes progressive restriction to an Osr1-dependent nephron progenitor compartment within the mammalian kidney. Dev Biol. 2008;324(1):88–98. Epub 2008/10/07. 10.1016/j.ydbio.2008.09.010 18835385 PMC2642884

[30] Xu J, Liu H, Park JS, Lan Y, Jiang R. Osr1 acts downstream of and interacts synergistically with Six2 to maintain nephron progenitor cells during kidney organogenesis. Development. 2014;141(7):1442–52. Epub 2014/03/07. 10.1242/dev.103283 24598167 PMC3957368

[31] Zhang Z, Iglesias D, Eliopoulos N, El Kares R, Chu L, Romagnani P, et al A variant OSR1 allele which disturbs OSR1 mRNA expression in renal progenitor cells is associated with reduction of newborn kidney size and function. Hum Mol Genet. 2011;20(21):4167–74. Epub 2011/08/09. 10.1093/hmg/ddr341 21821672

[32] Lozic B, Krzelj V, Kuzmic-Prusac I, Kuzmanic-Samija R, Capkun V, Lasan R, et al The OSR1 rs12329305 polymorphism contributes to the development of congenital malformations in cases of stillborn/neonatal death. Medical science monitor: international medical journal of experimental and clinical research. 2014;20:1531–8. 25164089 10.12659/MSM.890916 PMC4156340

[33] Kreidberg JA, Sariola H, Loring JM, Maeda M, Pelletier J, Housman D, et al WT-1 is required for early kidney development. Cell. 1993;74(4):679–91. Epub 1993/08/27. 8395349 10.1016/0092-8674(93)90515-r

[34] Armstrong JF, Pritchard-Jones K, Bickmore WA, Hastie ND, Bard JB. The expression of the Wilms' tumour gene, WT1, in the developing mammalian embryo. Mech Dev. 1993;40(1–2):85–97. Epub 1993/01/01. 8382938 10.1016/0925-4773(93)90090-k

[35] Barnes JD, Crosby JL, Jones CM, Wright CV, Hogan BL. Embryonic expression of Lim-1, the mouse homolog of Xenopus Xlim-1, suggests a role in lateral mesoderm differentiation and neurogenesis. Dev Biol. 1994;161(1):168–78. 7904966 10.1006/dbio.1994.1018

[36] Dressler GR, Douglass EC. Pax-2 is a DNA-binding protein expressed in embryonic kidney and Wilms tumor. Proc Natl Acad Sci U S A. 1992;89(4):1179–83. 1311084 10.1073/pnas.89.4.1179 PMC48412

[37] Dressler GR, Deutsch U, Chowdhury K, Nornes HO, Gruss P. Pax2, a new murine paired-box-containing gene and its expression in the developing excretory system. Development. 1990;109(4):787–95. 1977574 10.1242/dev.109.4.787

[38] Cong L, Ran FA, Cox D, Lin S, Barretto R, Habib N, et al Multiplex genome engineering using CRISPR/Cas systems. Science. 2013;339(6121):819–23. 10.1126/science.1231143 23287718 PMC3795411

[39] Wang H, Yang H, Shivalila CS, Dawlaty MM, Cheng AW, Zhang F, et al One-step generation of mice carrying mutations in multiple genes by CRISPR/Cas-mediated genome engineering. Cell. 2013;153(4):910–8. 10.1016/j.cell.2013.04.025 23643243 PMC3969854

[40] Zhou B, Ma Q, Rajagopal S, Wu SM, Domian I, Rivera-Feliciano J, et al Epicardial progenitors contribute to the cardiomyocyte lineage in the developing heart. Nature. 2008;454(7200):109–13. Epub 2008/06/24. 10.1038/nature07060 18568026 PMC2574791

[41] Brophy PD, Ostrom L, Lang KM, Dressler GR. Regulation of ureteric bud outgrowth by Pax2-dependent activation of the glial derived neurotrophic factor gene. Development. 2001;128(23):4747–56. Epub 2001/12/04. 11731455 10.1242/dev.128.23.4747

[42] Nishinakamura R, Matsumoto Y, Nakao K, Nakamura K, Sato A, Copeland NG, et al Murine homolog of SALL1 is essential for ureteric bud invasion in kidney development. Development. 2001;128(16):3105–15. Epub 2001/11/02. 11688560 10.1242/dev.128.16.3105

[43] Motamedi FJ, Badro DA, Clarkson M, Rita Lecca M, Bradford ST, Buske FA, et al WT1 controls antagonistic FGF and BMP-pSMAD pathways in early renal progenitors. Nature communications. 2014;5:4444 10.1038/ncomms5444 25031030

[44] Clark AT, Bertram JF. Molecular regulation of nephron endowment. The American journal of physiology. 1999;276(4 Pt 2):F485–97. 10198407 10.1152/ajprenal.1999.276.4.F485

[45] Hohenstein P, Hastie ND. The many facets of the Wilms' tumour gene, WT1. Hum Mol Genet. 2006;15 Spec No 2:R196–201. Epub 2006/09/22. 16987884 10.1093/hmg/ddl196

[46] Rauscher FJ 3rd. The WT1 Wilms tumor gene product: a developmentally regulated transcription factor in the kidney that functions as a tumor suppressor. FASEB J. 1993;7(10):896–903. 8393820

[47] Kreidberg JA, Hartwig S. Wilms' tumor-1: a riddle wrapped in a mystery, inside a kidney. Kidney Int. 2008;74(4):411–2. 10.1038/ki.2008.307 18670405

[48] Donovan MJ, Natoli TA, Sainio K, Amstutz A, Jaenisch R, Sariola H, et al Initial differentiation of the metanephric mesenchyme is independent of WT1 and the ureteric bud. Dev Genet. 1999;24(3–4):252–62. Epub 1999/05/14. 10322633 10.1002/(SICI)1520-6408(1999)24:3/4<252::AID-DVG8>3.0.CO;2-K

[49] Cebrian C, Asai N, D'Agati V, Costantini F. The number of fetal nephron progenitor cells limits ureteric branching and adult nephron endowment. Cell reports. 2014;7(1):127–37. 10.1016/j.celrep.2014.02.033 24656820 PMC4049224

[50] Liu Z, Chen S, Boyle S, Zhu Y, Zhang A, Piwnica-Worms DR, et al The extracellular domain of Notch2 increases its cell-surface abundance and ligand responsiveness during kidney development. Dev Cell. 2013;25(6):585–98. 10.1016/j.devcel.2013.05.022 23806616 PMC3710456

