# Supplementary figures and images for: Osr1 Interacts Synergistically with Wt1 to Regulate Kidney Organogenesis
Source: PLoS One. 2016 Jul 21;11(7):e0159597. doi: 10.1371/journal.pone.0159597 (PMC4956120; doi:10.1371/journal.pone.0159597)

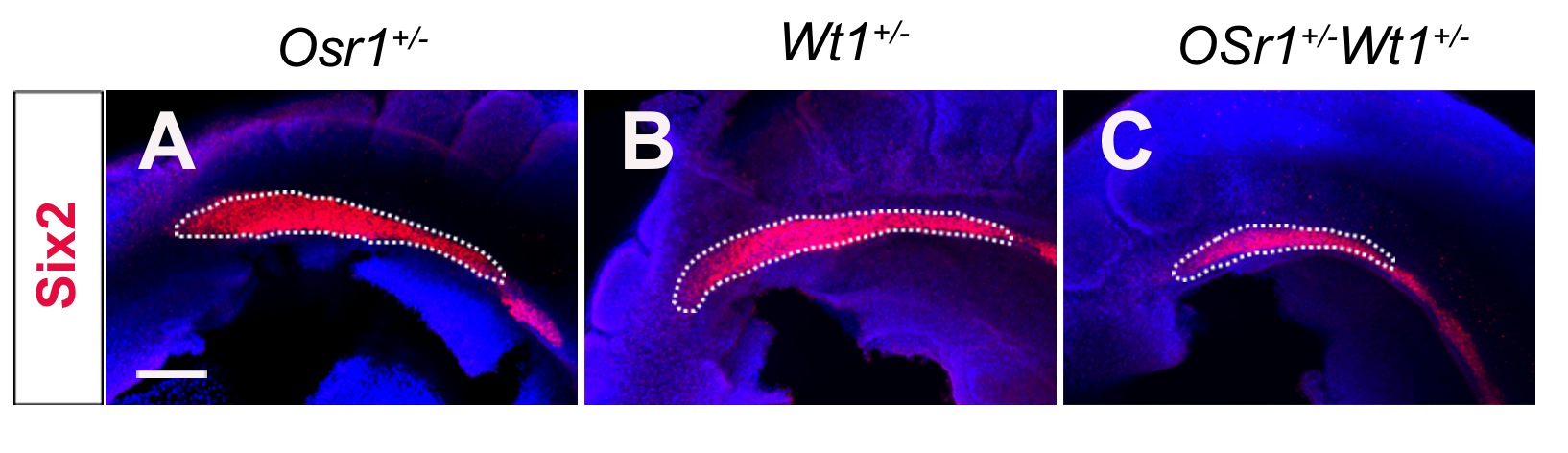

Supplement: S1 Fig — (A-C) Whole mount immunofluorescent staining for Six2 protein (red) in E10.5 Osr1+/- (A), Wt+/- (B), and Osr1+/-Wt1+/- (C) embryos. The embryos were counterstained with DAPI (Blue). The white dotted line outlines the metanephric mesenchyme. Scale bar, 100 μm. (TIF) [file pone.0159597.s001.tif]
